# Antiviral Activity against Avian Leucosis Virus Subgroup J of Degraded Polysaccharides from* Ulva pertusa*

**DOI:** 10.1155/2018/9415965

**Published:** 2018-08-05

**Authors:** Yuhao Sun, Xiaolin Chen, Lin Song, Song Liu, Huahua Yu, Xueqin Wang, Yukun Qin, Pengcheng Li

**Affiliations:** ^1^Key Laboratory of Experimental Marine Biology, Institute of Oceanology, Chinese Academy of Sciences, Qingdao 266071, China; ^2^Laboratory for Marine Drugs and Bioproducts of Qingdao National Laboratory for Marine Science and Technology, Qingdao 266071, China; ^3^University of Chinese Academy of Sciences, Beijing 100049, China; ^4^Center for Ocean Mega-Science, Chinese Academy of Sciences, Qingdao 266071, China; ^5^Qingdao University of Science and Technology, College of Marine Science and Biological Engineering, Qingdao 266042, China

## Abstract

Avian Leukosis Virus Subgroup J (ALV-J), a retrovirus of avian, has caused enormous economics losses to poultry industry around the world. Polysaccharides from marine algae are featured diversity bioactivities. To find the potential effect to prevent ALV-J spread, in this study, polysaccharides from* Ulva pertusa* (UPPs) and four low molecular weight (Mw)* U. pertusa* polysaccharides (LUPPs) were prepared and their functions on ALV-J were investigated. Firstly, LUPPs were obtained by hydrogen peroxide (H_2_O_2_) oxidative degradation. The effects of degradation conditions on Mw of the UPP were also investigated. Results showed that the H_2_O_2_ oxidative degradation method could degrade UPP effectively, and the degradation was positively related to H_2_O_2_ concentration and temperature and negatively to pH. The chemical characteristics of UPP and LUPPs were also determined. Afterwards, the anti-ALV-J activity of the polysaccharides were carried out in vitro. Results showed that UPP and LUPPs could inhibit ALV-J and LUPP-3 and Mw of 4.3 kDa exerted the strongest suppression. The action phase assay showed that LUPP-3 could bind with the viral particles and prevented ALV-J adsorption onto the host cells. And the ALV-J relative gene and gp85 protein expression were significantly suppressed after being administration with LUPP-3. Therefore, the low Mw polysaccharides from* U. pertusa* have great potential as an anti-ALV-J drug alternative.

## 1. Introduction

Avian Leukosis Virus Subgroup J (ALV-J) is an oncogenic retrovirus [1] which was first isolated in the UK in 1988 from meat-type chicken [2]. ALV-J often induced immunosuppression, high mortality, and growth retardation and leads to a variety of tumors such as hemangioma and myeloid leucosis [3, 4]. In the early to mid-1990s, an outbreak was observed in Japan, USA, Argentina, and other European countries, and it also spread to Malaysia and China by the early 2000s [5]. Although the eradication of breeding flocks against ALV-J was successful in some areas, the widespread distribution of ALV-J and the diverse culture model of poultry industry made it difficult to prevent and control ALV-J, which had caused severe economic losses in poultry industry worldwide [6, 7]. Until now, commercial vaccines and drugs are still not available against ALV-J infection [8, 9]. Therefore, it is necessary to find effective drugs or vaccines to control ALV-J spread.

Algae has received much attention because of the production of high-value products [10]. The products from the cell wall of marine algae include sulfated polysaccharides, such as fucoidan and carrageenan from brown and red seaweed, respectively [11].* Ulva* belongs to Family Ulvaceae, Class Chlorophyceae, and Chlorophyta Division [12]. Polysaccharide is one of the important bioactive compounds in* Ulva*. The mainly repeating disaccharide units of* Ulva* polysaccharide are [*β*-D-Glcp A-(1→4)-*α*-L-Rhap 3S] and [*α*-L-Idop A-(1→4)-*α*-L-Rhap 3S] [13]. And it has already proven to be a remarkable polysaccharide with multiple biological activity. For example, Qi et al. obtained several polysaccharides from* Ulva pertusa* and found that high sulfate content samples had more effective hydroxyl radical scavenging activity and stronger reducing power than natural ones [14]. Further, Thanh et al. isolated* U. lactuca* polysaccharides and demonstrated that the polysaccharide had a significant cytotoxic activity against three cancer cell lines: HepG2 (hepatocellular carcinoma), MCF7 (human breast cancer), and Hela (cervical cancer) [15]. In addition, in vitro and in vivo studies showed that the sulfated polysaccharides from* Ulva* exhibit anticoagulant, antibacterial, antiproliferative, antihyperlipidemic, and immune-modulatory activities [16–20]. Scientists also studied the antiviral activities of the polysaccharides from* Ulva*. Reports showed that the* Ulva* polysaccharide had anti-HSV-1, Japanese encephalitis virus, white spot syndrome virus, etc. [21–23]. However, the researches of* Ulva* polysaccharide focusing on poultry viruses were few.

The high molecular weight (Mw) of polysaccharides limited their application due to their physical properties such as low solution in water and high viscosity which decreased their biological activity [24]. In comparison, low Mw algae polysaccharide was degraded by various methods from high Mw polysaccharide [25–27] and it has higher water solubility and stability and easy organism absorption [28]. Recently, it was reported that the low Mw polysaccharides extracted from algae carried some effects on the inhibition of HIV, HSV-1, and H1N1 virus [29–31]. However, the studies aimed at finding the effects against ALV-J of algal polysaccharides were scarce. So, the applications of low Mw polysaccharides from* Ulva* in preventing ALV-J infection would be promising and innovative.

In our experiment,* U. pertusa *polysaccharides (UPPs) were extracted by traditional hot water extraction; then, hydrogen peroxide (H_2_O_2_) oxidative degradation method was used to prepare several low Mw* U. pertusa* polysaccharides (LUPPs) and the chemical composition of LUPPs was determined. Meanwhile, the effects of different degradation conditions (including pH, temperature, and H_2_O_2_ concentration) on Mw of UPP were also explored. Finally, the anti-ALV-J activity of LUPPs was tested in vitro. The results of this study would provide theoretical basis for exploration and development of algal polysaccharides as a new strategy in control of the ALV-J.

## 2. Materials and Methods

### 2.1. Reagents and Seaweeds Samples

*U. pertusa *was collected from the number 1 bathing beach of Qingdao, China, in April 2016. After washing with tap water, the algae was dried at 50°C to constant weight and stored at room temperature before being used. All reagents used were of analytical grade.

### 2.2. Extraction of UPP

UPP was extracted by hot water extraction and alcohol precipitation method reported by Zhang et al. [32] with some modification. Briefly, 30 g of dried algae was mixed with 1.2 L of distilled water and maintained at 125°C for 4 h. Then, the mixture was filtered and the supernatant was condensed by a rotary evaporator. The solution was dialyzed against distilled water for 48 h using dialysis tube with a 3.5 kDa Mw cut-off. After condensing again, 3-fold volume anhydrous ethanol was added to the dialyzed solution. The mixture was placed overnight at 4°C and then centrifuged. The precipitate was lyophilized and referred to as UPP. The yield (%) of the UPP was calculated by the formula: yield = (polysaccharides weight/algal dry weight).

### 2.3. Degradation of UPP

UPP was dissolved in distilled water in concentration of 20 mg/mL. The solutions were heated in a water bath with stirring at different H_2_O_2_ concentration and desired pH condition. Every 30 min, 0.5 mL of the reaction mixture was removed for high performance gel permeation chromatography (HPGPC) to investigate the effect of pH, H_2_O_2_ concentration, and temperature on the UPP degradation. Finally, according to the above results, LUPPs were prepared under different conditions.

### 2.4. Chemical Characterization

HPGPC method was used to measure the Mw of UPP and LUPPs with a TSK gel G3000PWxl column. The samples were detected by a refractive index detector, using 0.1 mol/L Na_2_SO_4_ as the mobile phase, and the flow rate was 0.5 mL/min also with a column temperature of 35°C. Dextran standards (Mw 1000, 5000, 12000, 50000, 80000, and 270000, Sigma, USA) were used to calibrate the column.

The protein content was analyzed by Bradford's method [33] using bovine serum albumin (BSA) as the standard. Phenol-sulfuric acid method [34] was used to detect the total sugar content and using rhamnose as the standard. Barium chloride gelatin method [35] was used to determine sulfate content. The FT-IR spectra of samples were measured in a KBr pellet by Nicolet-360 FT-IR spectrometer.

### 2.5. Cells, Virus, and Antibodies

The NX0101 strain of ALV-J, ALV-J gp85-specific monoclonal antibody, and DF-1 cell line were kindly gifted by Professor Cheng, Shandong Agricultural University. Dulbecco's modified Eagle's medium (DMEM) supplemented with 1% (v/v) or 10% (v/v) fetal bovine serum (FBS) was the maintenance medium (MM) or growth medium (GM), respectively. The ALV-J was titrated by quantifying the tissue culture infectious dose 50 (TCID_50_) using the Reed-Muench formula.

### 2.6. Cytotoxicity Test

The cytotoxicity of UPP and LUPPs on DF-1 cells was evaluated by an MTT (3-(4, 5-dimethyl-2-thiazolyl)-2,5-diphenyl-2-H-tetrazolium bromide) assay. Briefly, DF-1 cell monolayers grown in triplicate in 96-well plate were supplied with 100 *μ*L MM which contained the indicated polysaccharides (from 2 mg/mL to 0.03125 mg/mL), and the cells that were supplied with 100 *μ*L MM without polysaccharides were set as the cell control. The supernatant was removed after 24 h incubation; then the cell was added with 20 *μ*L MTT and maintained for another 4 h. Later, the MTT was discarded and 100 *μ*L DMSO was added. Subsequently, the absorbance was read at the wavelength of 490 nm using a plate reader (iMark™ Bio-Rad). The cytotoxic activity was calculated by the following equation: survival rate (%) = (Ae/Ac) ×100%, where Ae and Ac were the absorbance of experimental group (Ae) and cell control (Ac), respectively.

### 2.7. Antivirus Test In Vitro

#### 2.7.1. ALV p27 Antigen Detection

DF-1 cells seeded in 96-well plate were adsorbed with 100 TCID_50_ of ALV-J and mixed with the indicated polysaccharides dissolved in MM at a final concentration of 2 mg/mL for 2 h at 37°C. Next, the polysaccharides and the virus were washed away and the cells were covered with 2 mg/mL corresponding polysaccharides that dissolved in MM. Meanwhile, the DF-1 cells that were not infected with ALV-J and the cells that were not exposed to the polysaccharides were set as the cell control and virus control. After 24 h incubation at 37°C, the viral titers were measured by the ALV p27 antigen test kit (IDEXX, USA). The relative expressions of ALV p27 antigen were represented by the formula: S/P= (Sample mean-Negative control mean)/(Positive control mean-Negative control mean), and the negative control and positive control were provided by the kit.

#### 2.7.2. Polysaccharides Action Stage Assay

*Before Adsorption (BA)*. The MM containing 1 mg/mL polysaccharides was added to DF-1 cell monolayers and incubated for 2 h. Then, the cells were washed with PBS and inoculation with 100 TCID_50_ of ALV-J at 37°C for 2 h. After incubation, the unadsorbed viruses were washed away and the cells were covered with simple MM for 24 h.

*Adsorption (Ad)*. DF-1 cells were infected with 100 TCID_50_ of ALV-J and mixed with the indicated polysaccharides that dissolved in MM at a final concentration of 1 mg/mL. After incubation at 4°C for 2 h, the cells were washed with PBS and then incubated in MM for 24 h.

*After Adsorption (AA).* After being incubated with 100 TCID_50_ of ALV-J for 2 h, the DF-1 cells were washed by PBS and incubated with MM containing polysaccharides at a concentration of 1 mg/mL for 24 h.

Later, all supernatants were collected. And ALV p27 antigen test kit was used to determine the viral titers.

#### 2.7.3. ALV-J Gene Relative Expression and Protein Expression

DF-1 cells were seeded in 12-well plate, infected with 100 TCID_50_ of ALV-J, and mixed with the polysaccharides that dissolved in MM at indicated concentration for 2 h adsorption at 37°C. Then the cells were washed with PBS, covered by MM, and incubated at 37°C with 5% CO_2_ for about 24 h.

*Real-Time PCR*. Real-time PCR were used to determine the ALV-J gene relative expression in DF-1 cells. In brief, the total RNA of the treated DF-1 cells were extracted by RNAprep Pure Cell/Bacteria Kit (TIANGEN BIOTECH Co. Ltd., Beijing, China). Then PrimeScript™ RT reagent Kit and SYBR®* Premix ExTaq*™ Kit (Takara BIO Inc., Liaoning, China) were used for reverse transcription and real-time PCR. The forward and reverse primers for ALV-J were 5′-TGCGTGCGTGGTTATTATTTC-3′ and 5′-AATGGTGAGGTCGCTGACTGT-3′, respectively; and the forward and reverse primer for internal control GAPDH were 5′-GAACATCATCCCAAGCGTCCA-3′ and 5′-CGGCAGGTCAGGTCAACAAC-3′. The real-time PCR were performed at 95°C for 30 s, 95°C for 5 s and 60°C for 34 s (34 cycles), 95°C for 15 s, and 60°C for 60 s [36].

*Western Blot.* After 24 h incubation, the cells were lysed and protein concentrations were determined with a BCA protein assay kit (Beyotime Biotechnology). Protein samples were separated by SDS-PAGE and transferred to 0.45 *μ*m PVDF membrane (Millipore) for Western analysis. The membrane was blocked with 5% nonfat milk in TBST buffer (20 mM Tris-HCl, 500 mM NaCl, 0.1% Tween 20) for 1 h at room temperature and incubated with mouse ALV-J gp85-specific monoclonal antibody overnight at 4°C. Then, membrane was incubated with horseradish peroxidase- (HRP-) conjugated secondary antibodies for 1 h at room temperature and visualized using ECL reagents. The densities of the protein bands were normalized to that of *β*-tubulin, which served as an inner control.

*Indirect Immunofluorescence Assay (IFA)*. The DF-1 cells were incubated for 5 days at 37°C after inoculation. The cells were washed with PBS and added with cold acetone and ethanol (3:2 for v/v) for about 10 min and then washed with PBS again followed by being treated with the gp85-specific monoclonal antibody overnight at 4°C. Afterwards, the cells were washed thoroughly with PBS and incubated with goat anti-mouse IgG-FITC at 37°C for 1 h. After washing, the cells were directly observed using an inverted fluorescence microscope.

### 2.8. Statistical Analysis

SPSS were used to perform the statistical analysis and the differences between groups were analyzed by one-way ANOVA.

## 3. Results

### 3.1. Influence of Degradation Condition on the Mw of UPP

#### 3.1.1. Effect of pH

**Figure 1:**
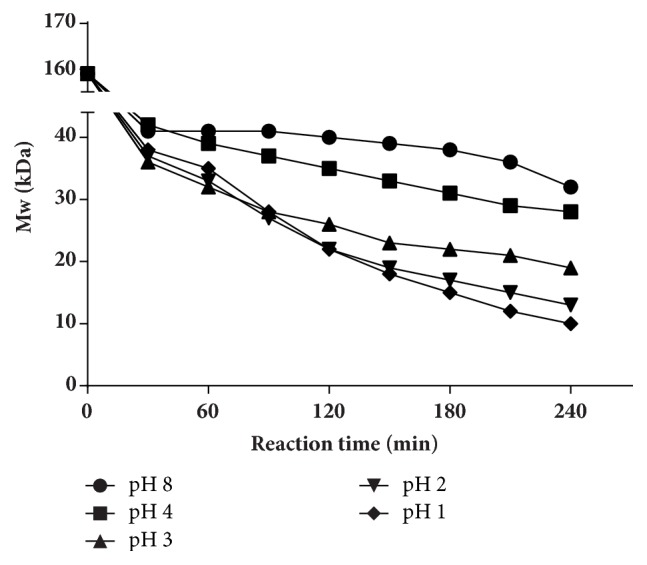
Effect of pH on degradation of UPP. UPP was degraded in 0.3%  H_2_O_2_ at 80°C and pH values of 1, 2, 3, 4, and 8 were tested.

As the results showed in Figure 1, in 30 min, the Mw was relatively close and decreased to 41 kDa and 42 kDa, respectively, when the pH was 4 and 8. But, after 30 min, the degradation for pH 8 groups was slower than pH 4 gradually. Compared with pH 4 and 8, before 90 min, the degradation curves were closer as well when pH decreased to 3, 2, and 1, and Mw of UPP was 28 kDa, 27 kDa, and 28 kDa at 90 min, respectively. Subsequently, degradation became different and the final degradation results were 10 kDa, 13 kDa, and 20 kDa, respectively. The results showed that the Mw of UPP was nearly the same at the beginning of the degradation. And the difference among different pH groups increased with the time going on. Generally, the lower pH is, the lower Mw polysaccharides were obtained.

#### 3.1.2. Effect of H_*2*_O_*2*_ Concentration

**Figure 2:**
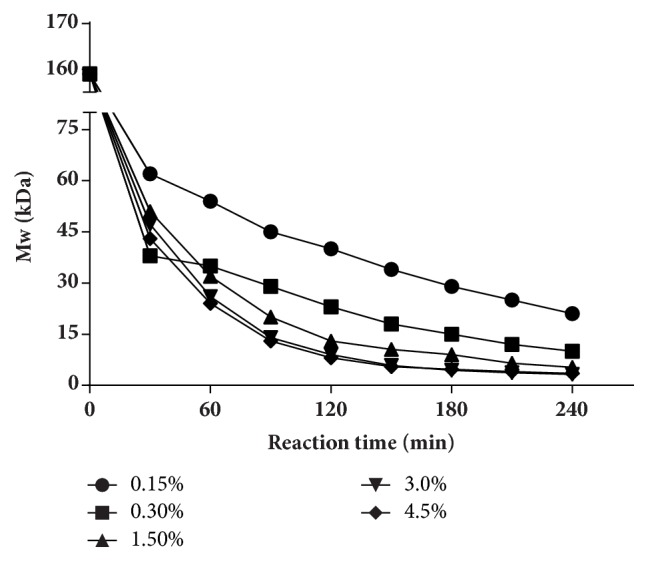
Effect of H_2_O_2_ concentration on degradation of UPP. The reaction temperature was 80°C and pH 4 was used. Different H_2_O_2_ concentrations varying from 0.15% to 4.5% were tested.

Figure 2 showed the Mw changes of UPP at different H_2_O_2_ concentration. The Mw changed to 54 kDa, 35 kDa, 32 kDa, 26 kDa, and 24 kDa corresponding to original H_2_O_2_ concentration of 0.15%, 0.3%, 1.5%, 3.0%, and 4.5% at 60 min, respectively. The Mw decreased with the degradation proceeding, and the final Mw values of the degraded polysaccharides were 21 kDa, 10 kDa, 5.3 kDa, 3.5 kDa, and 3.2 kDa, respectively. Moreover, the curves of 3.0% and 4.5% groups were similar from beginning to the end. The results illustrated that higher H_2_O_2_ concentrations were more favorable for degradation, and 3.0%  H_2_O_2_ was enough for degradation to obtain the low Mw polysaccharides.

#### 3.1.3. Effect of Temperature

**Figure 3:**
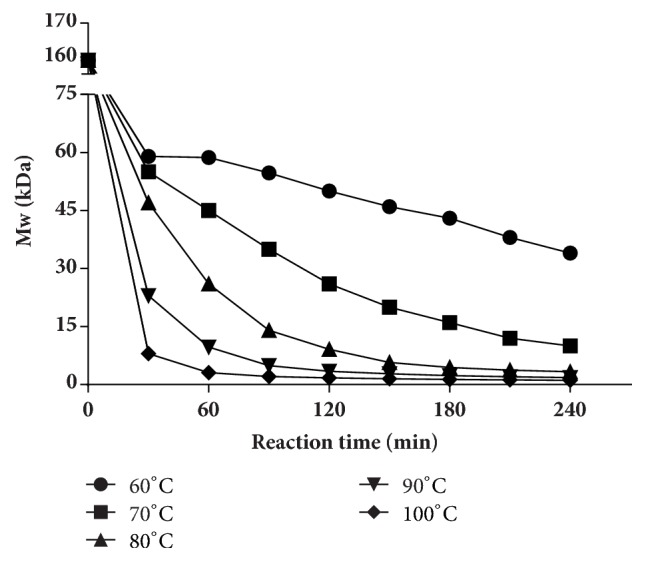
Effect of temperature on degradation of UPP. UPP was degraded in pH 4 and 3%  H_2_O_2_. Temperatures from 60°C to 100°C were tested.

Figure 3 showed the degradation of the UPP under different temperature conditions. When the temperature was 60°C, the Mw of the polysaccharide was 59 kDa at 30 min, and, finally, the Mw only decreased to 34 kDa, while the Mw of the polysaccharides in other four temperature groups showed more significant changes in the degradation process. For 70°C and 80°C, the Mw values were around 10 kDa and 3.3 kDa at 240 min, significantly lower than that of 60°C group. The Mw of the polysaccharides sharply decreased to 23 kDa and 8 kDa at 30 min when the temperatures were 90°C and 100°C; later, the degradation slowed down and the curves tended to stay with the polysaccharides Mw of 1.8 kDa and 1.1 kDa at last. The results revealed that higher temperature could accelerate the degradation.

### 3.2. Chemical Analysis and Preparation of LUPP

#### 3.2.1. Preparation of LUPP

According to the above results of H_2_O_2_ oxidative degradation on UPP, several kinds of LUPPs with different Mw were prepared by a series of conditions. After degradation, the LUPP solutions were neutralized to pH 7 and dialyzed in distilled water for about 48 h. Then the solutions were filtered and freeze-dried. The specific preparation conditions are presented in Table 1.

#### 3.2.2. Chemical Characterization

**Figure 4:**
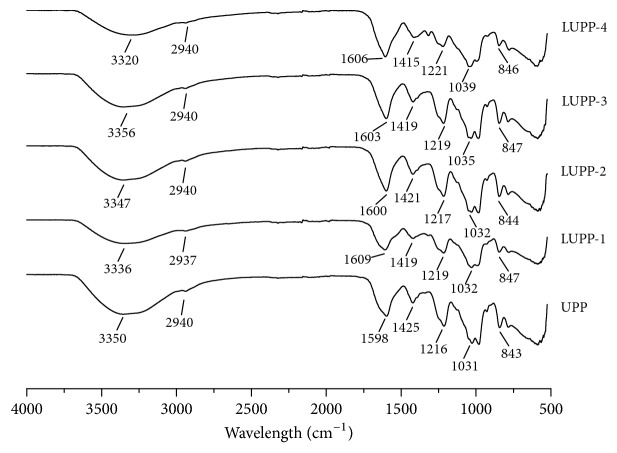
FT-IR spectra of UPP and LUPPs.

Based on the methods described in Section 3.2.1, four kinds of LUPPs were prepared. The chemical composition and FT-IR spectra of initial UPP and LUPPs are shown in Table 2 and Figure 4. The chromatograms of Dextran standards, UPP, and LUPPs are given in Figure S1-S11 in the Supplementary Materials. The HPLC standard curve equation was y = −2.4616x + 24.507 with the elution time as the ordinate and Log Mw as the abscissa. According to the above equation, the Mw of UPP and LUPPs were 159 kDa, 23.6 kDa, 9.0 kDa, 4.3 kDa, and 2.5 kDa, respectively. UPP possessed the highest total sugar content for about 44.61%, and the total sugar content decreased with the decrease of Mw. But the sulfate content was about 20% for all samples. UPP also had the highest protein content, only 1.12%, and the protein content decreased with the decrease of Mw among the samples.

Results of FT-IR spectra were shown in Figure 4. Typical absorption peaks at 3330 cm^−1^ and 2940 cm^−1^ represent O–H and C–H stretching vibration. The C=O asymmetric and symmetric stretching vibration appear at 1600 cm^−1^ and 1420 cm^−1^, respectively. Absorption at 1030 cm^−1^ indicates C–O–H deformation vibration. Featured absorption at 1220 cm^−1^ corresponds to S=O stretching vibration and the peaks at 845 cm^−1^ might reflect C–O–S symmetry stretching vibration [37, 38]. Generally speaking, the absorption peaks for all the groups were similar which indicated that the degradation did not significantly affect the chemical structure of the polysaccharides.

### 3.3. Cytotoxicity

MTT assay was applied to estimate the safe concentration of UPP and LUPPs. According to previous reports [39, 40], the polysaccharides were considered to have no cytotoxic activity when the cell survival rate is over 85%. As the results showed in Table 3, in general, all of the cell relative survival rates were over 95% at the concentration 2 mg/mL; therefore, 2 mg/mL of all the polysaccharides was still safe for further study.

### 3.4. Antiviral Activity

#### 3.4.1. ALV p27 Antigen Detection

**Figure 5:**
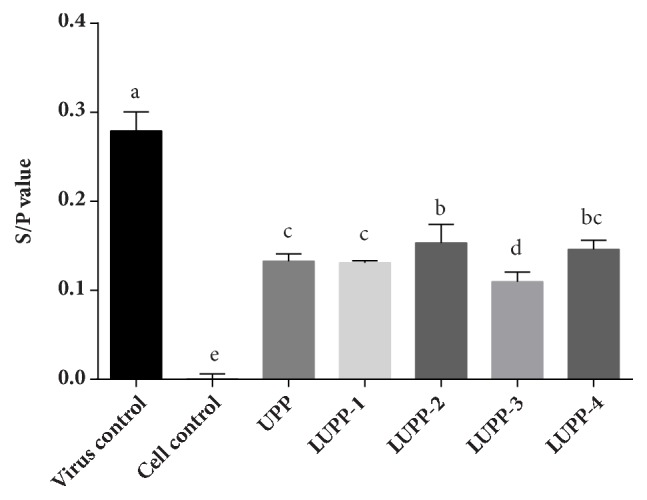
Relative expression of ALV p27 antigen. After being treated with ALV-J and polysaccharides for 2 h simultaneously, the cells were washed by PBS and incubated with MM with corresponding polysaccharides for 24 h. Values represent mean + standard deviation (n=3). Statistical significance p < 0.05, compared with each other.

Figure 5 showed the relative expression of ALV p27 antigen that was determined by ELISA method. The ALV p27 antigen expression was decreased for all the polysaccharides groups when treated with 2 mg/mL sample solutions, and it was significantly lower than that of the virus control. The S/P value of LUPP-2 was 0.153 and higher than that of the UPP (0.132) and LUPP-1 (0.131). In addition, the S/P value of LUPP-4 was not significantly different from the other experimental groups, except LUPP-3, indicating that the anti-ALV-J effect was not correlated with Mw simply. Among all the samples, the LUPP-3 with Mw of 4.3 kDa featured the lowest p27 relative expression, whose S/P value was only 0.110. Thus, LUPP-3 was cautiously chosen for further experiments.

#### 3.4.2. Action Phase of the Polysaccharides

**Figure 6:**
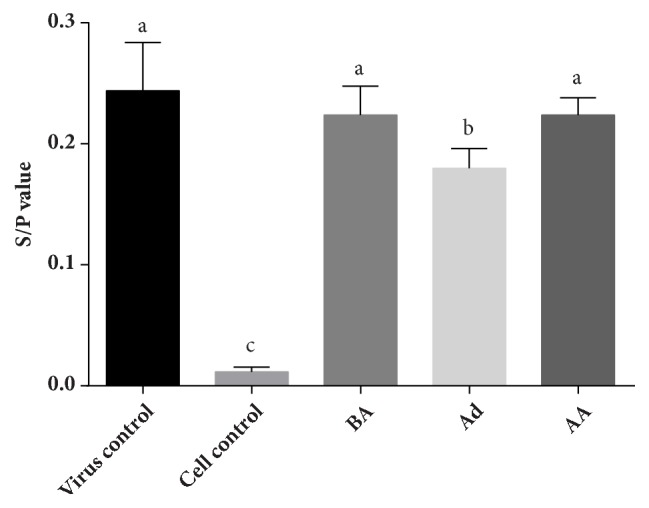
p27 expression of different modes of administration detected by ELISA. BA: DF-1 cells handled with polysaccharides before virus adsorption; Ad: DF-1 cells handled with polysaccharides at the virus adsorption phase; AA: DF-1 cells handled with polysaccharides after virus adsorption. Values represent mean + standard deviation (n=3). Statistical significance p < 0.05, compared with each other.

To explore the function time point of the polysaccharides, LUPP-3 was tested in different administration. And ELISA was used to determine the antiviral effect. The results presented in Figure 6 showed that the S/P values of BA (before adsorption) and AA (after adsorption) groups were both 0.224, which were not significantly lower than the virus control (0.244). However, the S/P value of Ad administration was 0.180, significantly lower than the virus control. Therefore, the above results indicated that the LUPP-3 could inhibit virus attachment to the cells; nevertheless, it could not take effect in the stage before virus adsorption or after the virus penetrated into the cells.

#### 3.4.3. RT-PCR Assay

**Figure 7:**
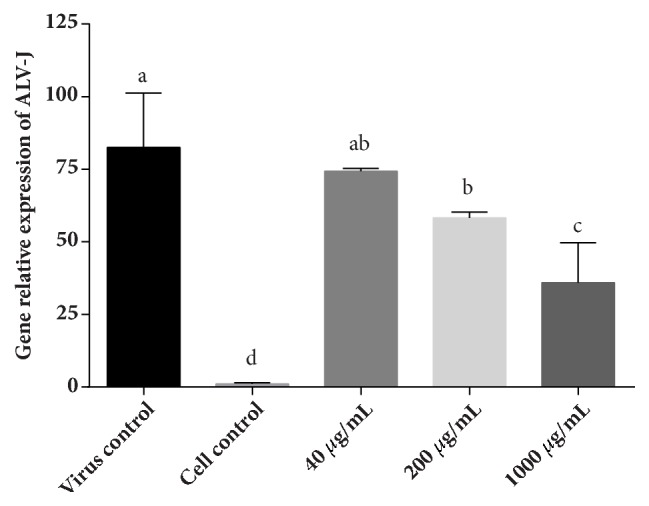
Gene relative expression of ALV-J. DF-1 cells inoculated with ALV-J and polysaccharides of different concentration for 2 h simultaneously. Then the cells were washed by PBS and covered with MM for 24 h. After that, the cells were collected for RT-PCR assay. Values represent mean + standard deviation (n=3). Statistical significance p < 0.05, compared with each other.

As the results illustrated in Figure 7, the gene relative expression of ALV-J decreased gradually with the increase of LUPP-3 concentration. Treatment with 1000 *μ*g/mL and 200 *μ*g/mL showed a strong suppression against ALV-J adsorption and the gene expression was 35.91 and 58.22, significantly lower than the virus control (82.49). However, when treated with 40 *μ*g/mL LUPP-3, the gene expression was 74.29, lower than the virus control but the difference was not significant. The results illustrated that LUPP-3 showed a better inhibition against ALV-J in a medium or high concentration, and it was not desirable in a low concentration.

#### 3.4.4. Determination of ALV-J gp85 Protein

**Figure 8:**
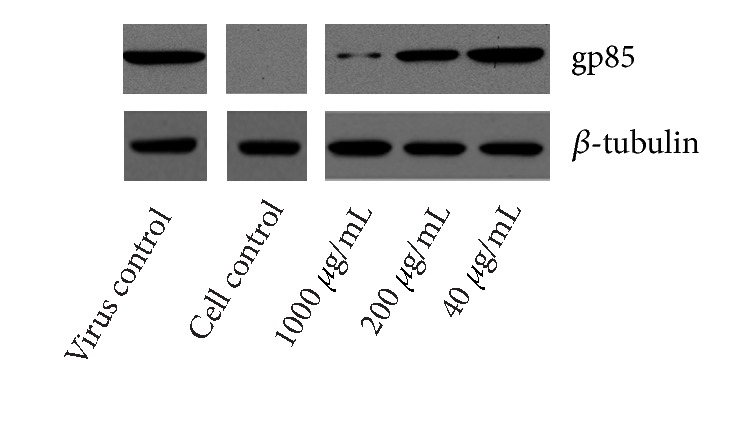
Western-blot analysis of ALV-J gp85 protein expression. DF-1 cells inoculated with ALV-J and polysaccharides of different concentration for 2 h simultaneously. Then the cells were washed by PBS and covered with MM for 24 h. After that, the cells were collected and lysed for western-blot assay.

To further verify the antiviral effect of LUPP-3, western blot and IFA were used to determine the protein expression of ALV-J. As the results of western blot showed in Figure 8, similar to the RT-PCT results, the antiviral effects were dose dependent. Expressions of gp85 protein were suppressed when administrated with 1000 *μ*g/mL and 200 *μ*g/mL LUPP-3, and the suppression of 1000 *μ*g/mL treatment group was more significant. While treating with 40 *μ*g/mL LUPP-3, the gp85 protein expression was similar to the virus control.

**Figure 9:**
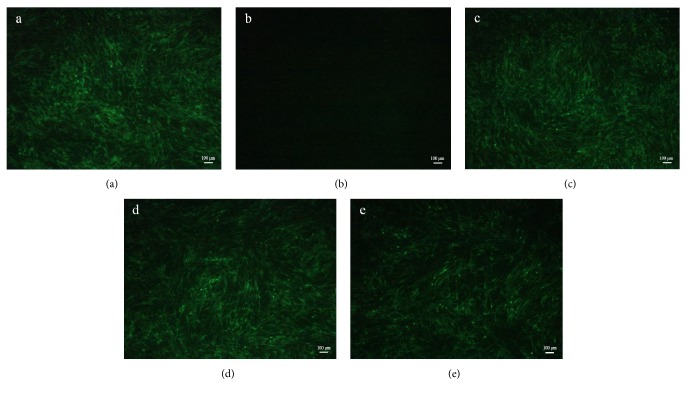
IFA analysis of ALV-J gp85 protein. DF-1 cells inoculated with ALV-J and polysaccharides of different concentration for 2 h simultaneously. Then the cells were washed by PBS and covered with MM for 5 days. After that, the IFA was carried out. (a) Virus control, (b) cell control, (c) ALV-J handled with 40 *μ*g/mL LUPP-3, (d) ALV-J handled with 200 *μ*g/mL LUPP-3, and (e) ALV-J handled with 1000 *μ*g/mL LUPP-3.

In IFA assay, the green fluorescence intensity could generally reflect the number of ALV-J. As the results showed in Figure 9, the fluorescence intensity of 40 *μ*g/mL group was the strongest, which was similar with the virus control. In addition, different from the RT-PCR and western-blot results, the 200 *μ*g/mL group also exhibited a strong fluorescence signal, which was the same with the results of virus control and 40 *μ*g/mL group. However, the fluorescence intensity of 1000 *μ*g/mL group was significantly weaker, which indicated that the ALV-J adsorption was effectively suppressed.

## 4. Discussion

Various methods are applied for degrading polysaccharides, such as chemical method [41], physical method [42], and enzymatic method [43] (Liu et al. 2017). Among them, the H_2_O_2_ oxidative degradation which belongs to chemical method is characterized by low cost and mild reaction, and it has been widely used in polysaccharides degradation. In the degradation process, the temperature, pH value, and H_2_O_2_ concentration were considered to have a great impact on the degradation. In this study, the degradation accelerated with the rising of the temperature and H_2_O_2_ concentration and with the decreasing of pH, in general. Results of the pH experiment showed that each group produced similar product in 30 min, and then the difference of the products' Mw between each group increased with the time going on. Eventually, the Mw of the products decreased with the decrease of pH. pH affects the generation rate of free radical, thus affecting the degradation rate [44]. At the same time, the acidic environment provided by the low pH may also directly degrade the polysaccharide chain. However, the Mw of the product could not decrease to lower than 10 kDa on pH 1 condition, and we speculated that it might be due to the low temperature and H_2_O_2_ concentration. H_2_O_2_ concentration is also an important factor that affects UPP degradation. For example, Lee et al. degraded *β*-glucan on 0.5 mM iron (II) sulfate heptahydrate (FeSO_4_·7H_2_O) at 50°C for 1 h and found that the Mw of the product was 561.2 kDa for 0.2%  H_2_O_2_ group, while the result decreased to 55.4 kDa with 1.0%  H_2_O_2_ [45]. H_2_O_2_ could degrade polysaccharides by producing free radicals that would interrupt glycosides randomly in aqueous solutions [46]. In our study, the degradation was positively related to H_2_O_2_ concentration from the beginning, which might be due to the more free radicals generated by the higher H_2_O_2_ concentration. But the results of 3.0% and 4.5% concentration groups were similar, which suggest that 3.0%  H_2_O_2_ was enough for degradation. Another important factor in degrading UPP was temperature. Higher temperature means greater kinetic energy, allowing more collisions between molecules [47]. As shown in Figure 3, temperature had a great impact on degradation. A product of 8 kDa could be obtained within 30 min at 100°C. But, for 80°C and 90°C groups, it needs more than 120 min and 60 min to get the same Mw product, respectively. In addition, 60°C and 70°C groups could not produce the 8 kDa product within 240 min, eventually. Therefore, increasing temperature significantly accelerated the degradation.

According to the degradation results above, we prepared four LUPPs and determined their chemical composition preliminarily. Results showed that the total sugar content decreased with the decrease of the Mw, and we inferred that it might be due to the comprehensive reaction caused by the temperature, H^+^, and free radicals. Meanwhile, the protein content also decreased along with the decrease of Mw, which indicated that the degradation might benefit for protein removal. Additionally, the sulfate content was nearly the same and around 20% for all samples. The FT-IR spectra showed that UPP and LUPPs had similar characteristic peak, which implied that the degradation did not significantly change the chemical structure of UPP. And the featured adsorption at 845 cm^−1^ and 1220 cm^−1^ indicated that all samples were sulfated algae polysaccharides.

Since it was found in 1988, ALV-J has caused enormous economic loss to poultry breeding industry across the world [48, 49]. However, because of the high mutation rate and the fact that the host factors that regulate viral infection are largely unknown [50], effective therapeutics are still not available for inhibiting ALV-J [8]. Eradicating the positive chickens was the only way to prevent ALV-J spread.

Until now, research on the anti-ALV-J activity of* Ulva* polysaccharides has not been reported. In this study, UPP and LUPPs were applied on ALV-J to investigate the antiviral effect for the first time. Results showed that the relative expression of ALV-J p27 antigen was significantly reduced on the presence of all samples. Effect of LUPP-3 with Mw of 4.3 kDa was the best, and the inhibition of LUPP-2 and LUPP-4 was weaker than that of UPP and LUPP-1, indicating that the antiviral activity was not only correlated with the Mw. Many studies have shown that the sulfate content of the polysaccharide has a great influence on its biological activities [51–53]. But, in our study, the sulfate content was nearly the same in all samples, and the antiviral effect exerted no relation with the total sugar content. Therefore, we deduced that the complicated suppression was probably caused by the comprehensive action that induced by Mw, sulfate, and some other factors. LUPP-3 might have a more suitable spatial structure to interact with the virus. Based on the above results, LUPP-3 was screened for subsequent experiments. To further explore the action stage of LUPPs, LUPP-3 was administrated in three different methods. Results revealed that the expression of p27 antigen decreased only with Ad treatment, which demonstrated that LUPP-3 could inhibit ALV-J adsorbtion on the host cells, and this effect might be achieved by the polysaccharides adsorption to the virus or to the cell surface specific receptors. Nevertheless, the polysaccharides had no inhibition when they were applied to DF-1 cells before inoculation (BA method), so we speculated that LUPP-3 could adsorb on the virus and form a virus-polysaccharides complex rather than adsorb onto the cell surface. The virus envelope sites which would contact with cell surface receptors were occupied by the polysaccharides, leading to the termination of virus invasion process. Some other types of algal polysaccharides were reported to act with the same mode as our samples. Carrageenan derivatives, for example, could bind to HSV virions by first changing the structure of the glycoprotein B (gB) and glycoprotein C (gC) of HSV, contributing to virus glycoprotein inactivation during the viral adsorption stage [54]. Saha et al. extracted a sulfated xylogalactofucan from* Laminaria angustata* and found that the polysaccharide had antiviral activity by direct interaction with viral particles [55], which might mask viral structures necessary for further interactions with host cells. However, research also showed that polysaccharides from* U. clathrata* could inhibit viral fusion by preventing the intact protein F0, which was essential for the syncytia formation of Newcastle Disease Virus (NDV), from being cleaved into the mature form [56]. Compared with our results, the disparate mechanism might be due to the different extraction sources that resulted in different polysaccharides structure, and the virus was also different. To further evaluate the antiviral effect of LUPP-3, RT-PCR, western blot, and IFA were used to determine the gene and protein expression of ALV-J. Expressions of ALV-J gene and gp85 protein were observed to be significantly reduced when treated with 200 *μ*g/mL and 1000 *μ*g/mL LUPP-3 in RT-PCR and western-blot experiments. The results indicated that the ALV-J adsorption was suppressed and thus inhibited the subsequent life process. Results of IFA were different from that of RT-PCR and western blot, and expression of gp85 protein only reduced when handled with 1000 *μ*g/mL LUPP-3. It was probably because the DF-1 cells in IFA experiment were maintained for five days after inoculation and much longer than the one day in the other two experiments. After being treated with 200 *μ*g/mL LUPP-3 and inoculation, the ALV-J that successfully adsorb on DF-1 cells could be fully replicated, resulting in similar results compared with the viral control. However, the question of how LUPP binds to the virus surface and whether it could be specifically attached to the virus proteins which would combine with the cell receptor needs to be further illuminated in the future.

## 5. Conclusion

Initial polysaccharides from* U. pertusa* were degraded by H_2_O_2_ oxidative degradation. The effects of degradation conditions on the degradation process were explored; at the same time, four LUPPs were prepared. In addition, chemical composition of UPP and LUPPs was also characterized. Finally, the anti-ALV-J activity of all samples was investigated. Results showed that H_2_O_2_ oxidative degradation could degrade UPP effectively, and the degradation was positively related to H_2_O_2_ concentration and temperature and negatively to pH. The Mw of UPP and four LUPPs was 159, 23.6, 90, 4.3, and 2.5 kDa, respectively, and the degradation did not change the chemical structure of UPP. The antiviral experiment revealed that UPP and four LUPPs could inhibit ALV-J in vitro; among them, LUPP-3 possessed the strongest anti-ALV-J effect. Subsequent study showed that LUPP-3 could bind with the viral particles and prevented ALV-J adsorption on the host cells. Further experiment indicated that LUPP-3 could reduce the infection probability of ALV-J and contributed to the significant decrease of ALV-J gene and protein expression. However, the function mechanism between LUPPs and ALV-J should be further researched. This study enhanced our understanding of LUPPs and gave guidance for its potential use in the future.

## Figures and Tables

**Table 1:** Degradation conditions and yield of LUPPs.

Sample	Temperature (°C)	pH	H_2_O_2_ (%)	Time (min)	Yield (%)
LUPP-1	70	4	3	45	58.22
LUPP-2	80	4	3	125	39.02
LUPP-3	90	4	3	90	17.60
LUPP-4	100	4	3	90	22.65

**Table 2:** Properties of the polysaccharides (%w/w of dry weight). Values are as mean ± standard deviation (n=3).

Sample	Mw (kDa)	Total Sugar (%)	Sulfate (%)	Protein (%)
UPP	159 ± 0.9	44.61 ± 1.20	17.46 ± 0.24	1.12 ± 0.01
LUPP-1	23.6 ± 0.5	42.49 ± 0.65	21.24 ± 0.16	0.76 ± 0.01
LUPP-2	9.0 ± 0.2	38.77 ± 0.69	19.43 ± 0.06	0.19 ± 0.01
LUPP-3	4.3 ± 0.3	37.59 ± 0.44	20.18 ± 0.24	0.17 ± 0.01
LUPP-4	2.5 ± 0.1	33.40 ± 0.35	20.63 ± 0.23	0.28 ± 0.01

**Table 3:** Relative Survival Rate of DF-1 cells. Values are as mean ± standard deviation (n=3).

Concentration (mg/mL)	2	1	0.5	0.25	0.125	0.0625	0.03125
UPP	0.96 ± 0.02	1.03 ± 0.04	1.02 ± 0.03	1.09 ± 0.05	1.01 ± 0.06	0.96 ± 0.04	1.06 ± 0.04
LUPP-1	0.95 ± 0.01	1.09 ± 0.02	1.00 ± 0.04	1.01 ± 0.05	0.96 ± 0.07	1.10 ± 0.04	1.04 ± 0.06
LUPP-2	0.94 ± 0.04	1.09 ± 0.03	1.07 ± 0.01	1.08 ± 0.05	1.05 ± 0.02	0.97 ± 0.03	1.06 ± 0.04
LUPP-3	0.95 ± 0.03	1.11 ± 0.05	1.04 ± 0.05	1.07 ± 0.01	1.11 ± 0.08	0.96 ± 0.07	1.11 ± 0.04
LUPP-4	0.99 ± 0.03	0.98 ± 0.06	1.06 ± 0.04	0.98 ± 0.02	1.09 ± 0.05	1.04 ± 0.04	0.99 ± 0.05

## Data Availability

The authors applied for a patent for the content of this study and so it cannot be made freely available. Access to these data will be considered by the authors upon request, with the permission of Xiaolin Chen.

## Supplementary Materials

Supplementary Materials Figure  S1: chromatograms of Dextran standards with Mw of 1000. Figure  S2: chromatograms of Dextran standards with Mw of 5000. Figure  S3: chromatograms of Dextran standards with Mw of 12000. Figure  S4: chromatograms of Dextran standards with Mw of 50000. Figure  S5: chromatograms of Dextran standards with Mw of 80000. Figure  S6: chromatograms of Dextran standards with Mw of 210000. Figure  S7: HPLC profile of UPP. Figure  S8: HPLC profile of LUPP-1. Figure  S9: HPLC profile of LUPP-2. Figure  S10: HPLC profile of LUPP-3. Figure  S11: HPLC profile of LUPP-4.
