# Supplementary material for: Antiviral Activity against Avian Leucosis Virus Subgroup J of Degraded Polysaccharides from Ulva pertusa
Source: Biomed Res Int. 2018 Aug 5;2018:9415965. doi: 10.1155/2018/9415965 (PMC6098872; doi:10.1155/2018/9415965)
Supplement: Supplementary Materials — Figure S1: chromatograms of Dextran standards with Mw of 1000. Figure S2: chromatograms of Dextran standards with Mw of 5000. Figure S3: chromatograms of Dextran standards with Mw of 12000. Figure S4: chromatograms of Dextran standards with Mw of 50000. Figure S5: chromatograms of Dextran standards with Mw of 80000. Figure S6: chromatograms of Dextran standards with Mw of 210000. Figure S7: HPLC profile of UPP. Figure S8: HPLC profile of LUPP-1. Figure S9: HPLC profile of LUPP-2. Figure S10: HPLC profile of LUPP-3. Figure S11: HPLC profile of LUPP-4. [file 9415965.f1.docx]

**Supplementary material**

FIGURE S1. Chromatograms of Dextran standards with Mw of 1000.

FIGURE S2. Chromatograms of Dextran standards with Mw of 5000.

FIGURE S3. Chromatograms of Dextran standards with Mw of 12000.

FIGURE S4. Chromatograms of Dextran standards with Mw of 50000.

FIGURE S5. Chromatograms of Dextran standards with Mw of 80000.

FIGURE S6. Chromatograms of Dextran standards with Mw of 210000.

FIGURE S7. HPLC profile of UPP.

FIGURE S8. HPLC profile of LUPP-1.

FIGURE S9. HPLC profile of LUPP-2.

FIGURE S10. HPLC profile of LUPP-3.

FIGURE S11. HPLC profile of LUPP-4.
